# Deep Radiotranscriptomics of Non-Small Cell Lung Carcinoma for Assessing Molecular and Histology Subtypes with a Data-Driven Analysis

**DOI:** 10.3390/diagnostics11122383

**Published:** 2021-12-17

**Authors:** Eleftherios Trivizakis, John Souglakos, Apostolos Karantanas, Kostas Marias

**Affiliations:** 1 Computational Biomedicine Laboratory (CBML), Foundation for Research and Technology Hellas (FORTH), 70013 Heraklion, Greece; souglak@uoc.gr (J.S.); karantanas@uoc.gr (A.K.); kmarias@ics.forth.gr (K.M.); 2 Medical School, University of Crete, 71003 Heraklion, Greece; 3 Laboratory of Translational Oncology, Medical School, University of Crete, 71003 Heraklion, Greece; 4 Department of Medical Oncology, University Hospital of Heraklion, 71500 Heraklion, Greece; 5 Department of Radiology, Medical School, University of Crete, 71003 Heraklion, Greece; 6 Department of Electrical and Computer Engineering, Hellenic Mediterranean University, 71410 Heraklion, Greece

**Keywords:** non-small cell lung carcinoma, radiotranscriptomics, deep features, radiomics, transcriptomics, machine learning, multi-view learning

## Abstract

Radiogenomic and radiotranscriptomic studies have the potential to pave the way for a holistic decision support system built on genomics, transcriptomics, radiomics, deep features and clinical parameters to assess treatment evaluation and care planning. The integration of invasive and routine imaging data into a common feature space has the potential to yield robust models for inferring the drivers of underlying biological mechanisms. In this non-small cell lung carcinoma study, a multi-omics representation comprised deep features and transcriptomics was evaluated to further explore the synergetic and complementary properties of these diverse multi-view data sources by utilizing data-driven machine learning models. The proposed deep radiotranscriptomic analysis is a feature-based fusion that significantly enhances sensitivity by up to 0.174 and AUC by up to 0.22, compared to the baseline single source models, across all experiments on the unseen testing set. Additionally, a radiomics-based fusion was also explored as an alternative methodology yielding radiomic signatures that are comparable to several previous publications in the field of radiogenomics. Furthermore, the machine learning multi-omics analysis based on deep features and transcriptomics achieved an AUC performance of up to 0.831 ± 0.09/0.925 ± 0.04 for the examined molecular and histology subtypes analysis, respectively. The clinical impact of such high-performing models can add prognostic value and lead to optimal treatment assessment by targeting specific oncogenes, namely the response of tyrosine kinase inhibitors of *EGFR* mutated or predicting the chemotherapy resistance of *KRAS* mutated tumors.

## 1. Introduction

The highest mortality rate worldwide has been estimated as being among lung cancer patients, according to a recent report [1] by the World Health Organization (WHO [2]). Therapeutic decisions for non-small cell lung carcinoma (NSCLC) in contemporary clinical practice are based on empirical observations of clinicians in association with histological, genomic, clinical, laboratory and other routine imaging data [3]. Gene expression analysis provides insights into the biological functions and molecular structure of neoplasms, while the profiling of specific malignancies contributes to the discovery of novel and discriminative biomarkers by selecting an optimal and personalized treatment [4]. The molecular characteristics of NSCLC should be considered in treatment decisions as they are involved in the crucial mechanisms of lesion progression [5]. Furthermore, the effectiveness of radiomics is based on the hypothesis that medical image analysis can quantify the underlying disease. In this context, radiogenomic/radiotranscriptomic analysis [6] has two main goals: (a) the correlation of imaging with genomic/transcriptomic features, and (b) the combination of the aforementioned data sources to improve robustness for increased predictive power. In particular, the accurate prediction of the genetic alterations of targeted oncogenes has a high clinical significance in precision medicine as they have the potential to uncover prognostic drivers for treatment response [7,8,9,10,11,12,13,14,15,16].

NSCLC radiogenomic/radiotranscriptomic analyses in the current literature mainly focus on predicting molecular and histological subtypes, solely from imaging data, and correlating genomic signatures with radiomic features [17,18,19,20,21,22,23,24,25,26]. Only a handful of studies have combined selected radiomic and transcriptomic features into a unified predictive signature. In particular, radiotranscriptomics of adipose tissue has been used for risk assessment in cardiovascular disease [27]. In oncology, Chaddad et al. [28] performed a variety of multi-omics analyses by integrating radiomics with genomics, transcriptomics, proteomics and clinical data to assess the survival rate of IDH1 wild-type glioblastoma patients. Fan et al. [29] implemented a nomogram-based integration of radiomics, transcriptomics and clinical parameters to estimate the objective response rate, and the overall and progression-free survival of NSCLC patients treated with radiotherapy. Thus, the combination of the two data sources provided a robust and improved model in terms of predictive power [28,29].

In this study, a multi-view analysis was used to combine deep features with selected transcriptomic features in a common multi-omics space to predict the molecular subtypes (*EGFR*, *KRAS* mutation) and histological subtypes (adenocarcinoma or squamous cell carcinoma) of NSCLC patients. Applying domain agnostic and data-driven machine learning techniques to the examined deep radiotranscriptomic data has the advantage of capturing the biological variability of NSCLC and, consequently, improving robustness and prediction performance. Despite the increased popularity of artificial intelligence, to the best of our knowledge, the current work is the first deep radiotranscriptomic analysis of NSCLC that integrates selected transcriptomics and deep features into a unified feature space.

## 2. Materials and Methods

### 2.1. Dataset

The NSCLC Radiogenomics [30] dataset comprises 211 CT routine examinations in total, with 142 available ePad [31] pixel-based lesion annotations and 211 image markup standards (AIM files), and an additional 162 PET/CT examinations, 130 RNA-seq vectors (P_G_) and clinical data with genomic, histology, semantic, survival or disease recurrence information. The patient cohort of this study includes up to 112 subjects from the examined database, specifically the routine CT scans with the available pixel-based annotations, transcriptomic data and well-defined histology or molecular endpoints. The aforementioned clinical data includes patients with characterizations such as *EGFR* mutation status (L*_EGFR_* = 172), *KRAS* mutation status (L*_KRAS_* = 171) and histology subtype (L_HS_ = 211). A subset of 142 CT examinations has available annotations on a pixel basis for the region of interest (P_ROI_). The intersection of the imaging and transcriptomic data, denoted by P_ROI_ ∩ P_T_, is a set of 115 patients (P_RG_). The final cohorts of *EGFR* (P*_EGFR_* = L*_EGFR_* ∩ P_RG_ = 92), *KRAS* (P*_KRAS_* = L*_KRAS_* ∩ P_RG_ = 93) and histology (P_HS_ = L_HS_ ∩ P_RG_ = 112) subtypes were considered for the proposed radiotranscriptomic analyses.

### 2.2. Multi-View Learning for Radiotranscriptomics

**Figure 1:**
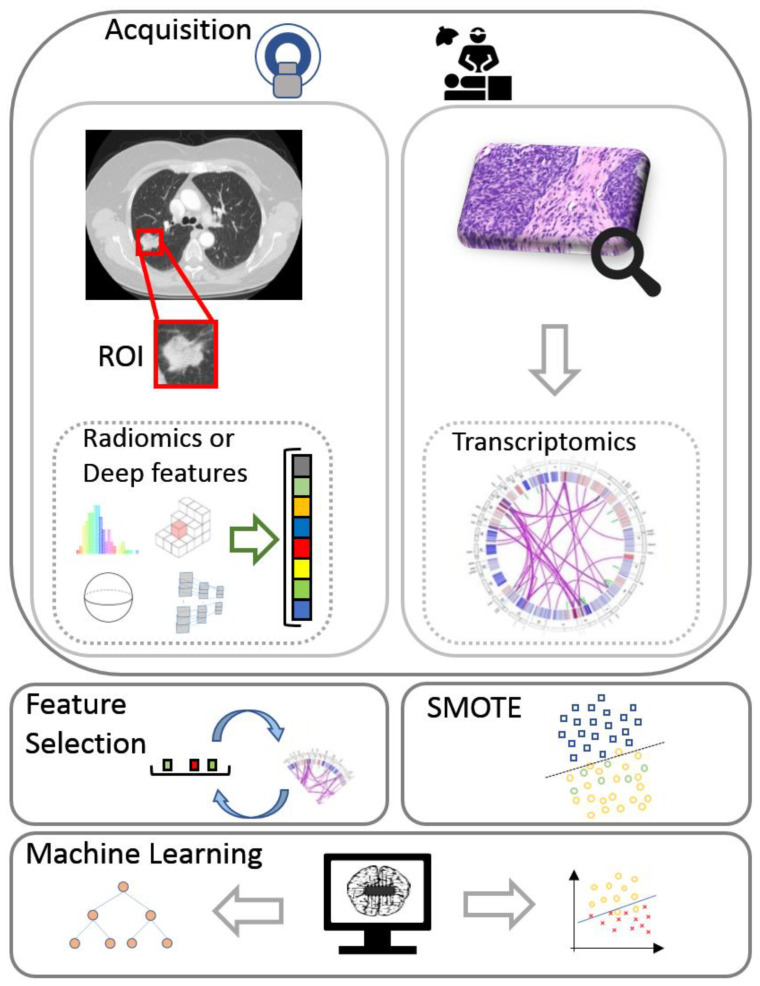
The flow diagram of the proposed radiotranscriptomic analysis incorporates acquisition of transcriptomics and computed tomography data with pixel-wise lesion delineation, followed by feature extraction, feature selection, minority oversampling, multi-view integration, and machine learning analysis. ROI, region of interest; SMOTE, synthetic minority oversampling technique.

Two data views were considered in this study: (a) deep features or traditional radiomics and (b) transcriptomics. The fusion strategy that was incorporated into the proposed radiotranscriptomic analysis includes the concatenation of both views into a common feature space prior to classification. A depiction of the full radiotranscriptomic pipeline can be found in Figure 1. Details regarding the selected parameters and the complete source code of the analysis are provided online [32].

#### 2.2.1. Deep Features

Deep learning has a substantial impact on image analysis tasks, primarily because of the deep models’ capacity to generalize [33,34]. This is achieved by learning low-level filters that are incorporated into the hierarchical inner representation of the convolutional part of the deep model [35]. Transfer learning (TL) is one of the most critical methodologies in deep learning since it enables the implementation of deeper models without the need for big data availability. Two main types of domain adaptation [36,37] have been proposed: (a) “off-the-shelf” TL, where the feature extraction part of a source model is transferred to the target model; and (b) fine-tuning TL, where the whole or part of the source model is transferred to the new model but the internal representation has to be adapted with a new training process. The latter methodology is more demanding on the dataset samples for model fitting, validation and evaluation. Thus, the “off-the-shelf” TL was used in the proposed methodology because of the low number of available samples in the examined patient cohort and the unbalanced natural prevalence of the disease. Additionally, this methodology has been successfully integrated into many medical image classification tasks, such as interstitial lung disease [37], colonic polyps [38], breast cancer [39], breast density assessment [40] and brain neoplasms [41], and evaluated across multiple other histopathology datasets [36].

The proposed “off-the-shelf” TL strategy incorporates pretrained ImageNet [42] models extracting raw deep features from the last convolution layer of the source model. Thus, no training was applied to the TL models since the investigated dataset was limited by size, which rendered de novo network development impractical. Eighteen models with a variety of architectures and parameters were used, including the most popular VGG [43], Inception [44], Xception [45], ResNet [46], NasNet [47], MobileNet [48] and DenseNet [49], and their variants that are available in the Keras [50] online repository. All pretrained convolutional layers were transferred to the new model, but the high level fully connected classification layers were removed to allow for the deep feature extraction from the low-level filters. The fully convolutional model was used on a per slice premise and the maximum pooling on a patient basis, resulting in a single compact representation of the three-dimensional volume of interest. Furthermore, the extraction was performed on the zero-padded CT region of interest (ROI) with a size of 150 by 150 pixels. Image normalization was performed prior to the padding. Depending on the architecture used, the number of raw features extracted per slice varied from 1088 to 65,919. Features with zero variance were removed, significantly reducing the length of the extracted vector, and feature standardization was applied for zero mean and unit variance before the analysis.

#### 2.2.2. Radiomics

The radiomic analysis comprised 2996 imaging features extracted with a fixed bin size from the volume of interest of the original CT examination. Shape features (fourteen in total) including elongation, flatness, sphericity, 3D and 2D diameter, mesh, surface and voxel volume were calculated in addition to the first order features (eighteen) of skewness, energy, entropy, kurtosis and other statistical features. Texture features (seventy-five), such as autocorrelation, cluster prominence, contrast, gray-level covariance (GLCM), dependence (GLDM), run length (GLRLM), size zone (GLSZM) and neighborhood gray-tone difference (NGTDM) matrix features, were extracted by the PyRadiomics framework [51] version 2.2.0. Additionally, isotropic resampling was performed using the built-in Pyradiomics preprocessing methods to achieve uniform spacing across patients. Other image filtering techniques (six) were applied to the original examination, including exponential, gradient, Laplacian of Gaussian, square, square root and wavelet filtering (twenty-two) prior to feature extraction, which enriched the proposed radiomic analysis by augmenting the final feature vector. In particular, mother wavelets such as daubechies, symlets, coiflets, biorthogonal and reverse biorthogonal with decomposition up to the second level were applied to the original examination.

#### 2.2.3. Transcriptomics

Transcriptomic data provide details about carcinogenesis procedures and neoplasm progression [52]. Additionally, transcriptomic profiling is a significant technology for improving diagnosis, patient stratification and the identification of prognostic biomarkers [4]. Thus, this personalized transcriptomic evaluation could promote bespoke therapies or response prediction based on the specific neoplasm composition. The examined RNA-seq data were downloaded from the NCBI GEO hosting database [53]. The pseudonyms for the subjects are the same as those used in the image database. In total, 130 RNA-seq vectors were available for radiotranscriptomic analysis. The original transcriptomics comprised 22,126 values but, after removing incomplete features, a transcriptomic signature of 5268 molecules per patient was examined.

#### 2.2.4. Feature Selection

The analysis of variance (ANOVA) was used separately as an objective-specific feature selection process for transcriptomics and deep feature vectors. This supervised univariate method reduced the dimensionality of the extracted feature vectors in a meaningful way according to the examined classification objective during the experimental phase. The resulting statistically significant components were selected for identifying potential markers for differentiating between the mutation status and histology subtypes of the examined patients. A combined analysis of *p* values with respect to their corresponding F scores for transcriptomic, radiomic and deep features assisted in the selection of a subset of the most significant radiotranscriptomic features. Additionally, an L1 penalty powered by a linear regression model was applied to further reduce the feature space of each data view by minimizing the coefficients, which yielded a sparse representation. The extensive lists of both transcriptomic and radiomic signatures are provided in the Supplementary Materials.

#### 2.2.5. Synthetic Minority Oversampling Technique

A common problem in data analysis is that datasets contain unequal distributions across categories, with “normal” examples outnumbering the uncommon “abnormal” occurrences. This can lead to a negative bias with reduced sensitivity towards the minority class in machine learning classification. The sample generation with SMOTE [54] is a supervised approach that uses the k-nearest neighbors in the feature space to augment the samples with artificial data points. Between two neighbors, a new feature vector is generated and multiplied by a random factor of positive decimal with a value of less than one. SMOTE was applied in the training set to alleviate the imbalances during the model convergence. Additionally, experiments without the SMOTE were performed, maintaining the natural prevalence of the disease. The trained models were evaluated exclusively on “real” and unseen samples.

#### 2.2.6. Data Stratification

**Figure 2:**
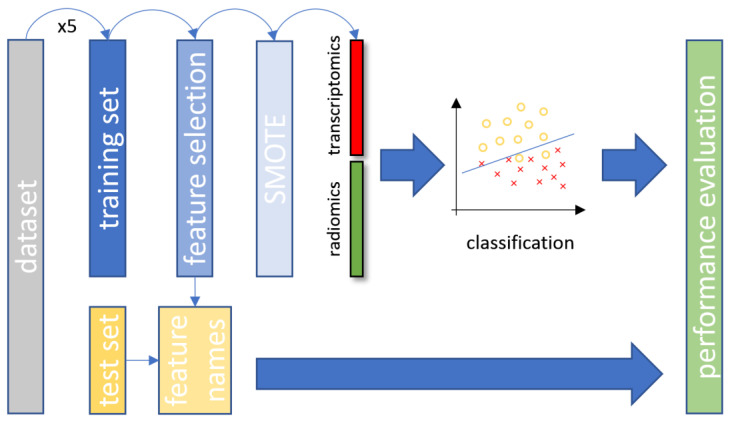
The overall data analysis process with the proposed CT and transcriptomic feature fusion in a combined machine learning analysis. SMOTE, synthetic minority oversampling technique; CT, computed tomography.

Fivefold cross-validation on a patient basis was applied to the original dataset for splitting into training and testing sets. Furthermore, for the examined patient cohort, patient stratification was applied in a way that preserved the balance of each class. The training set was used for model fitting, feature selection and oversampling of the minority class. The class distribution across the corresponding experiments was: (a) 80% wild-type and 20% mutant, (b) 76.1% wild-type and 23.9% mutant and (c) 79.8% adenocarcinoma and 20.2% squamous cell carcinoma. A key factor of the utmost importance during the experimental process was that the testing set remain unseen until the final stage of the performance evaluation, as depicted in Figure 2. This approach was applied to enhance the reliability for all developed machine learning models and avoid the overfitting of the data distribution or sample selection biases.

#### 2.2.7. Classification

Three binary tumor characterizations were examined: (a) EGFR mutation status, (b) KRAS mutation status and (c) histology subtypes. Seven classifiers were employed interchangeably for differentiating among the radiotranscriptomic, transcriptomic and radiomic signatures, namely: (a) k-NN; (b) decision tree; (c) RBF-GPC; (d) RBF-SVM; (e) linear SVM; (f) polynomial SVM; and (g) sigmoid SVM. The classifier implementations of the Sci-Kit Learn library [55] were used in this study.

## 3. Results

The analysis was performed on a computational node integrating an AMD Ryzen central processing unit with thirty-two threads, sixty-four gigabytes of random access memory and an RTX 3090 graphics processing unit with twenty-four gigabytes of video memory. Overall, the same experimental protocol and data stratification methodology were applied across all experiments, with the key differentiating factors being the feature fusion (radiomics or deep features with transcriptomics), oversampling technique and classifier. In total, 2394 unique models were evaluated, including deep radiotranscriptomics (756, SMOTE/not by deep models by endpoints by classifier types), traditional radiotranscriptomics (42, SMOTE/not by endpoints by classifier types), single source transcriptomics (798) and imaging models (798).

**Figure 3:**
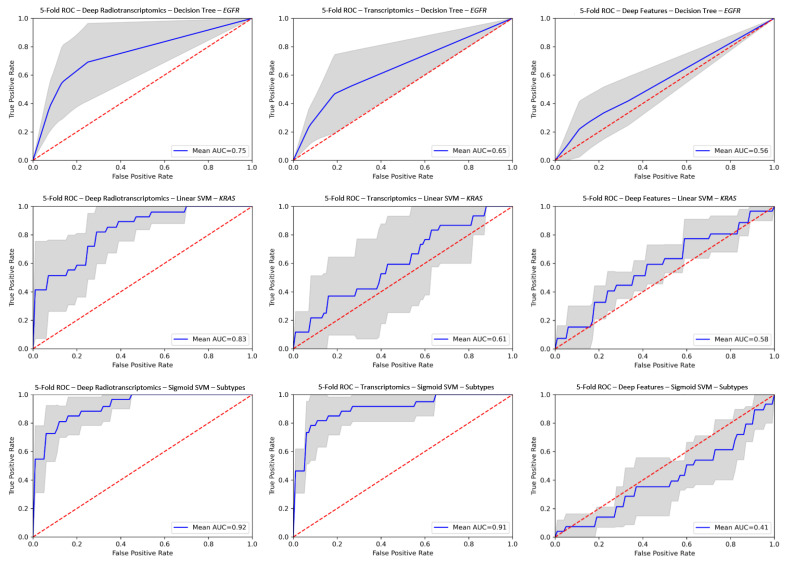
ROC curves for deep radiotranscriptomics (**left column**), transcriptomics (**center column**) and deep feature (**right column**) analysis. The top row represents EGFR (SMOTE), the middle row is KRAS and the bottom row is histology subtypes. The gray region represents the prediction variability among the unseen testing folds. AUC, area under curve; ROC, receiver operating characteristic; EGFR, epidermal growth factor receptor; KRAS, Kristen rat sarcoma; SVM, support vector machine; SMOTE, synthetic minority oversampling technique.

The comparison of the corresponding radiotranscriptomic against single source models on ROC curves in Figure 3 reveals the performance advantage in favor of the former, with significantly improved robustness and consistency throughout the examined experiments. In particular, improved performance was observed mainly in radiotranscriptomics for molecular and histological subtype differentiation compared to the best corresponding single source models with gains in AUC scores ranging from 0.016 to 0.22 (Tables S1–S4). The proposed radiotranscriptomics methodology achieved the best classification score with an AUC of 0.943 ± 0.03 on the histology subtype characterization with a linear SVM (Table 1). The deep radiotranscriptomics performed better in molecular subtypes, where the combination of the two sources of selected features impacted greatly on the prediction of *KRAS* mutation status (AUC 0.831 ± 0.09), with improved performance and increased prediction stability from the best single source transcriptomic model (AUC 0.611 ± 0.22). It should be noted that the pre-trained ResNet and DenseNet model families provided the best deep features. Additionally, it is worth mentioning that the single source deep features model for assessing *EGFR* and *KRAS* mutation status outperformed their counterparts in traditional radiomic models.

The inclusion of SMOTE in the analysis considerably improved both deep and traditional radiotranscriptomics for *EGFR* expression prediction, especially in terms of sensitivity. In particular, the traditional radiotranscriptomics achieved an AUC of 0.726 ± 0.10 (Table S2) over 0.645 ± 0.12 (Table S1) and the deep feature-based counterpart achieved an AUC of 0.747 ± 0.14 (Table S4) over 0.634 ± 0.24 (Table S3).

The traditional radiotranscriptomics for *EGFR* performed slightly better (AUC 0.645 ± 0.12 versus 0.642 ± 0.11) than the best single source counterpart, according to Table S1. This difference was negligible for the aforementioned model, with the prediction AUC only increasing by 0.003 compared to the transcriptomics model. Although, in terms of sensitivity and specificity, the difference is more pronounced with a difference of 0.04 and 0.043, respectively.

The most discriminative components in the radiomic signature for *EGFR* include features that reflect similarity, homogeneity, heterogeneity and complexity in texture patterns (gldm_DependenceNonUniformityNormalized, glszm_ZoneEntropy, glcm_MCC (maximal correlation coefficient), ngtdm_Strength). The *KRAS* radiomic signature comprises features that quantify skewness, asymmetry, local homogeneity and substantial variations in intensity values in the region of interest (glcm_ClusterShade, glcm_Idmn (inverse difference moment normalized), gldm_LargeDependenceLowGrayLevelEmphasis, ngtdm_Complexity). The radiomics of histology subtypes are mainly zone-based features that estimate the variability of zone size, the ratio of large and small zones to high gray-levels and skewness in texture complexity (glszm_SizeZoneNonUniformityNormalized, glszm_ZonePercentage, glszm_LargeAreaHighGrayLevelEmphasis, glszm_SmallAreaHighGrayLevelEmphasis, glcm_MCC), along with a few first order features such as the total energy, 90th percentile and wavelet minimum. It is important to note that at least half of the features in each radiomic signature were based on the wavelet filtered image. Furthermore, two detailed lists of the most discriminative features for both transcriptomics and radiomics in molecular or histological subtypes are shown in Tables S5 and S6. Detailed performance metrics of all the experiments are presented in Table 1 and Tables S1–S4 and the corresponding ROC curves in Figure 3 and Tables S2–S6.

## 4. Discussion

Artificial intelligence has advanced into an essential methodology for inferring knowledge from a high dimensional space with a data-driven perspective in many disciplines. In medicine, the increasing quantities of information could outline the complexity of the underlining biology of specific lesions, especially in oncology. While several efforts have used single source data to investigate and model cancer mechanisms [56,57,58,59,60], our effort is towards the synergistic use of high dimensional and high throughput data (deep features, radiomics and transcriptomics) for identifying the prognostic signatures towards precision decision support in oncology.

### 4.1. Common Features Found in Current Literature

The proposed approach yielded distinct signatures for *EGFR*, *KRAS* and histology subtypes across all three experiment types, as illustrated in Tables S5 and S6. Notably, the most significant radiomic feature in this analysis for *KRAS* mutation was the ngtdm_Complexity, which is the same feature found by Zhang et al. [61] for the same radiogenomic objective in a completely different patient cohort, indicating feature stability and robustness. Additionally, in the same study, the best radiomic feature for *EGFR* mutation assessment was the gldm_LargeDependenceHighGrayLevelEmphasis, which belongs to the same texture family as gldm_DependenceNonUniformityNormalized and is similar in nature to the high level feature glrlm_LongRunHighGrayLevelEmphasis, both also found in the present analysis. Furthermore, another agreement with the findings (Table S6) of the proposed analysis was observed for discriminative features of histology subtypes with studies reporting cluster shade [62], first-order, GLCM, GLSZM [63] and a combination of high level emphasis and small area emphasis [64] as important features. It is worth noting that the majority of the identified radiomics were wavelet-based features (Table S6), indicating that a significant part of the differentiating information exists only in specific frequency bands and can be deciphered through scale-space wavelet analysis [65].

### 4.2. Performance of Radiotranscriptomics Versus Single Source Models

The single source radiomics and deep features analyses achieved a performance at the lower end of the spectrum in terms of sensitivity, but both have shown increased specificity compared to the corresponding transcriptomic analyses. The deep features achieved a higher sensitivity in *EGFR*/*KRAS* mutation status assessment than the corresponding radiomics. The prediction of the molecular and histology subtypes was enhanced by the integration of imaging with transcriptomic data in a common feature space. The proposed radiotranscriptomics established models with an improved sensitivity of up to 0.182 compared to their single source counterparts, a superior AUC performance in mutation status prediction of up to 0.831 ± 0.09 and a histological subtype prediction of up to 0.942 ± 0.03. These results showcase the radiotranscriptomic synergy assumption between the two different sources of data, as discussed in the introduction. Furthermore, the benefits of radiotranscriptomic analysis can be summarized into the overall improved ROC curves (Figure 3), AUC score and sensitivity, and the reduced prediction variability in classification across all types of experiments (*EGFR*, *KRAS* and histology subtypes). In comparison, similar studies on imaging only data demonstrate significantly lower performance for *KRAS* mutation prediction, up to AUC 0.667 [23], and histological subtype differentiation ranging from 0.754 [21] to 0.893 [26]. In contrast, the radiotranscriptomics of *EGFR* did not improve on the current state-of-the-art study of Rizzo et al. [23], which was based solely on semantic CT features. This can also be attributed to the imaging modality, since better and more discriminative features or biomarkers [66] have been reported in several studies [25,67,68,69] where the PET/CT radiomic signature with an AUC of 0.805 significantly outperformed the CT only features (AUC 0.667) in *EGFR* mutation status differentiation. Despite the limited number of patients for machine learning analysis, especially regarding the molecular subtype patient cohorts, the proposed radiotranscriptomic model for *KRAS* differentiation outperformed the model of Rizzo et al. [23]. Additionally, the proposed ML-based analysis for molecular and histological subtypes outperformed the corresponding NSCLC state-of-the-art research [17,19,20,21,22,23,25,26] by a wide margin. The performance of the proposed radiotranscriptomics and the state-of-the-art literature is presented in detail in Table 2. The complete results of the radiotranscriptomic analyses are presented in Table 1 and Tables S1–S4 of the Supplementary Materials.

### 4.3. Clinical Impact of the Study

The proposed analyses in this study identified discriminative compact transcriptomics, radiomics and deep feature signatures to accurately model the underlying biology of non-small cell lung cancer. A multi-view learning methodology for high dimensional and low sample sized datasets [70] is essential for the integration of the different types of omics data. Two views that include deep features and transcriptomics were analyzed using distinct pipelines and were integrated prior to classification, with the purpose of capturing the heterogeneity of NSCLC. The high performance of the proposed deep radiotranscriptomics in assessing the genetic alterations of *EGFR* and *KRAS* oncogenes, as presented in the results section, could potentially add precision in treatment planning with TKIs or other targeted therapies.

Some challenges for single source RNA-seq data that significantly affect the generalization of the analysis are the varying data acquisition protocols [71], intratumor heterogeneity [72] and local mutation burden [73]. These are prominent aspects of non-small cell lung carcinomas. Transcriptomics is subjected to local mutational diversity, which can be as high as interpersonal variation [73]. Imaging features, on the other hand, are computed throughout the whole tumor, yielding complex signatures that include patterns from the microenvironment (necrotic, hypoxic and oxygenated tissue) of the neoplasm. Radiotranscriptomics has the potential to accurately capture the total mutational burden (TMB) by combining locally (transcriptomics) and globally (radiomics) dependent patterns. Therefore, the proposed composite signature addresses the shortcoming of single source data by assembling a holistic representation that fuses markers related to biological mechanisms (via transcriptomics) and tumor heterogeneity patterns (via imaging features extracted from the volume of interest). This can be beneficial for assessing clinically impactful endpoints, such as survival and therapy response [73]. To this end, we provide an open source repository [32] of the proposed methodology to encourage further research and to enable reproducibility and comparison with future studies.

### 4.4. Limitations and Future Extensions

A significant limitation of this study was the reduced data variability from an ethnically diverse patient cohort that reflects the genetic heterogeneity in non-small cell lung carcinomas. This is a key factor for enhancing the predictive power and robustness of radiotranscriptomic models. Another issue was the limited simultaneous availability of both imaging and transcriptomic data. The small dataset size increased prediction variability in many experiments, particularly in some transcriptomics and radiomics for molecular subtype models. The standardization, robustness and reproducibility of radiomics is an important issue in AI analysis that requires multiple examinations of the studied patient cohort. Nonetheless, CT scans use Hounsfield Units to capture the tissue’s electron density inside a particular voxel, which is a constant value for each tissue type, offering a unique radiation absorption signature and making CT quantitative by nature. The variability in the genetic and phenotypic profile of NSCLC tumors, which makes radiomic and transcriptomic analysis difficult, was another limiting factor. This can be observed in Table 1 where the standard deviation of the experiments can reach up to 0.22 in terms of AUC variability. As a result, parameter optimization in the machine learning methods that was used was especially challenging since it was difficult to assess the impact of the tuning. Therefore, the default parameters were used for every component of the pipeline, despite the feature type. Additionally, instead of a unified feature space, fusion could be achieved at the decision level with different classifiers or analysis pipelines for each data type. Finally, another limiting factor was the lack of the multi-modal fusion of imaging features because, while PET/CT examinations are available in this dataset, calculating radiomic and deep features was not feasible due to the lack of pixel-based annotations. The latter requires at least two expert clinicians to perform the delineation and achieve a consensus in terms of tumor margins.

Lastly, future research with a multi-institutional and independent patient cohort is a necessary step to evaluate the current experimental procedure by offering increased variety in the examined data sources through integrating different imaging equipment and genome extraction protocols. Additionally, an extension of the proposed data analysis methodology will be explored on the same patient cohort, including endpoints that rely on statistically depended clinical variables, such as lesion recurrence, overall survival or novel therapy-related markers, in order to adapt the therapeutic strategy [74,75] according to the patient’s radiogenomic profile into a personalized diagnosis and treatment plan. Other important molecular indicators for assessing targeted treatments include *ALK* rearrangements, *BRAF* mutation status and programmed death-ligand 1 (*PDL1*) expression, which will be investigated in a future radiogenomic study.

## 5. Conclusions

The deep radiotranscriptomics framework achieved state-of-the-art performance and, most importantly, improved classification metrics, such as AUC and sensitivity, compared to the baseline single source models in the examined molecular and histology subtype analyses. The proposed machine learning selection and feature fusion provided significant evidence supporting the hypothesis presented in the introduction regarding the complementary nature of the two feature types in the context of radiotranscriptomics. A closer collaboration between physicians and data scientists is essential for developing a trustworthy and explainable AI framework that aims to minimize erroneous diagnosis and optimize the planning of a personalized treatment strategy.

## Figures and Tables

**Table 1:** Performance of the ML-based pipeline on deep radiotranscriptomics and traditional radiotranscriptomics. The highest overall score between experiments is presented in bold.

Experiments	Classifier	Feature Type	Over-Sampling	ACC	AUC	SN	SPC
*EGFR*	Decision Tree	ResNet	SMOTE	**0.805 ± 0.05**	**0.747 ± 0.14**	**0.627 ± 0.33**	**0.869 ± 0.06**
*KRAS*	Linear SVM	DenseNet	No	**0.865 ± 0.08**	**0.831 ± 0.09**	**0.512 ± 0.25**	**0.974 ± 0.03**
Histology Subtypes	Sigmoid SVM	ResNet	No	0.888 ± 0.07	0.925 ± 0.04	0.743 ± 0.16	0.933 ± 0.06
*EGFR*	Sigmoid SVM	Radiomics-based	SMOTE	0.761 ± 0.10	0.726 ± 0.10	0.600 ± 0.16	0.800 ± 0.11
*KRAS*	Linear SVM		No	0.730 ± 0.05	0.719 ± 0.07	0.34 ± 0.27	0.883 ± 0.08
Histology Subtypes	Linear SVM		No	**0.907 ± 0.05**	**0.943 ± 0.03**	**0.797 ± 0.12**	**0.941 ± 0.03**

**Table 2:** The corresponding literature of the examined dataset with varying methodologies including semantic CT features, radiomic and radiotranscriptomics analyses (AUC). The highest overall score for each experiment type is presented in bold.

	*EGFR*	*KRAS*	Histological Subtypes
Proposed Traditional Radiotranscriptomics	0.726 ± 0.10	0.719 ± 0.07	**0.942 ± 0.03**
Proposed Deep Radiotranscriptomics	**0.747** **±** **0.14**	0.831 ± 0.09	0.924 ± 0.04
Morgado et al. [17]	0.737	-	-
Moreno et al. [19]	up to 0.82	up to 0.778	-
Dong et al. [20]	0.751	0.696	-
Yamada et al. [21]	-	-	0.754
Koyasu et al. [22]	0.659	-	0.843
Rizzo et al. [23]	0.823	0.667	-
Li et al. [25]	0.667	-	-
Zhu et al. [26]	-	-	0.893

## Data Availability

The examined computed tomography and transcriptomic dataset titled “NSCLC Radiogenomics” is available online as an open-access repository via the following link: https://wiki.cancerimagingarchive.net/display/Public/NSCLC+Radiogenomics (accessed on 31 March 2019).

## Supplementary Materials

The following are available online at https://www.mdpi.com/article/10.3390/diagnostics11122383/s1: Figure S1, Spearman’s correlations (rho > 0.6) among radiomic and transcriptomic features. No statistically significant radiogenomic correlations were found; Figure S2, ROC curves for radiotranscriptomics- (left column), transcriptomics- (center column) and radiomics-based (right column) analysis. The top row represents EGFR (SMOTE), the middle row is KRAS and the bottom row is histology subtypes. The gray region represents the prediction variability among the unseen testing folds. AUC, area under curve, ROC, receiver operating characteristic, EGFR, epidermal growth factor receptor, KRAS, Kristen rat sarcoma, SVM, support vector machine, SMOTE, synthetic minority oversampling technique; Figure S3, ROC curves for radiotranscriptomics- (left column), transcriptomics- (center column) and radiomics-based (right column) analysis. The top row represents EGFR, the middle row is KRAS and the bottom row is histology subtypes; Figure S4, ROC curves for SMOTE radiotranscriptomics- (left column), transcriptomics- (center column) and radiomics-based (right column) analysis. The top row represents EGFR, the middle row is KRAS and the bottom row is histology subtypes; Figure S5, ROC curves for deep radiotranscriptomics- (left column), transcriptomics- (center column) and deep descriptor-based (right column) analysis. The top row represents EGFR, the middle row is KRAS and the bottom row is histology subtypes; Figure S6, ROC curves for SMOTE deep radiotranscriptomics- (left column), transcriptomics- (center column) and deep descriptor-based (right column) analysis. The top row represents EGFR, the middle row is KRAS and the bottom row is histology subtypes; Table S1, Performance of the ML-based pipeline on CT 3D radiomics, transcriptomics and radiotranscriptomics analysis. The highest overall score is presented in bold; Table S2, Performance of the SMOTE ML-based pipeline on CT 3D radiomics, transcriptomics and radiotranscriptomics analysis. The highest overall score is presented in bold; Table S3, Performance of the ML-based pipeline on deep descriptors, transcriptomics and deep radiotranscriptomics analysis. The highest overall score is presented in bold; Table S4, Performance of the SMOTE ML-based pipeline on deep descriptors, transcriptomics and deep radiotranscriptomics analysis. The highest overall score is presented in bold; Table S5, The most significant transcriptomic features; Table S6, The most significant radiomic features.
