# Supplementary material for: Deep Radiotranscriptomics of Non-Small Cell Lung Carcinoma for Assessing Molecular and Histology Subtypes with a Data-Driven Analysis
Source: Diagnostics (Basel). 2021 Dec 17;11(12):2383. doi: 10.3390/diagnostics11122383 (PMC8700168; doi:10.3390/diagnostics11122383)
Supplement: Supplementary file 1 [file diagnostics-11-02383-s001.zip › diagnostics-1437887-SI.pdf]

diagnostics

Supplementary Materials: Deep Radiotranscriptomics of Non-Small Cell Lung Carcinoma for Assessing Molecular and Histology Subtypes with a Data-Driven Analysis

Eleftherios Trivizakis, Ioannis Souglakos, Apostolos H. Karantanas and Kostas Marias

Tables

Traditional Radiotranscriptomics:

Table S1. Performance of the ML-based pipeline on CT 3D radiomics, transcriptomics and radiotranscriptomics analysis. The highest overall score is presented in bold.

Experiments	Classifier	Feature type	ACC	AUC	SN	SPC
EGFR	Sigmoid SVM	Radiotranscriptomics	**0.820±0.07**	**0.645±0.12**	**0.527±0.15**	0.888±0.08
		Transcriptomics	0.776±0.08	0.642±0.11	0.487±0.08	0.845±0.10
		Radiomics	0.800±0.02	0.627±0.10	0.04±0.08	**0.989±0.02**
KRAS	Linear SVM	Radiotranscriptomics	**0.730±0.05**	**0.719±0.07**	**0.34±0.27**	0.883±0.08
		Transcriptomics	0.697±0.04	0.703±0.04	0.23±0.20	0.852±0.06
		Radiomics	0.726±0.06	0.437±0.19	0.09±0.11	**0.903±0.07**
Histology Subtypes	Linear SVM	Radiotranscriptomics	**0.907±0.05**	**0.943±0.03**	**0.797±0.12**	**0.941±0.03**
		Transcriptomics	0.896±0.04	0.932±0.04	0.757±0.08	0.939±0.04
		Radiomics	0.799±0.08	0.715±0.10	0.427±0.24	0.892±0.06

Table S2. Performance of the SMOTE ML-based pipeline on CT 3D radiomics, transcriptomics and radiotranscriptomics analysis. The highest overall score is presented in bold.

Experiments	Classifier	Feature type	ACC	AUC	SN	SPC
EGFR	Sigmoid SVM	Radiotranscriptomics	**0.761±0.10**	**0.726±0.10**	**0.600±0.16**	0.800±0.11
		Transcriptomics	0.759±0.10	0.677±0.13	0.533±0.19	**0.813±0.10**
		Radiomics	0.557±0.03	0.549±0.07	0.530±0.13	0.567±0.07
KRAS	Decision Tree	Radiotranscriptomics	**0.664±0.11**	**0.603±0.15**	**0.493±0.22**	0.713±0.09
		Transcriptomics	0.635±0.09	0.502±0.15	0.256±0.28	**0.747±0.09**
		Radiomics	0.505±0.06	0.428±0.05	0.287±0.12	0.569±0.09
Histology Subtypes	Sigmoid SVM	Radiotranscriptomics	**0.847±0.07**	**0.907±0.05**	**0.643±0.34**	**0.917±0.05**
		Transcriptomics	0.821±0.06	0.871±0.05	0.543±0.19	0.908±0.03
		Radiomics	0.713±0.07	0.642±0.06	0.100±0.2	0.864±0.08

Deep Radiotranscriptomics:

Table S3. Performance of the ML-based pipeline on deep descriptors, transcriptomics and deep radiotranscriptomics analysis. The highest overall score is presented in bold.

Experiments	Classifier	Deep Model (Layers)	Feature type	ACC	AUC	SN	SPC
EGFR	Sigmoid SVM	ResNetV2 (101)	Deep Radiotranscriptomics	0.768±0.06	0.634±0.24	0.250±0.25	0.907±0.08
		-	Transcriptomics	**0.807±0.13**	**0.712±0.26**	**0.407±0.27**	**0.910±0.09**
		ResNetV2 (101)	Deep Features	0.757±0.08	0.595±0.19	0.23±0.16	0.892±0.08
KRAS	Linear SVM	DenseNet (169)	Deep Radiotranscriptomics	**0.865±0.08**	**0.831±0.09**	**0.512±0.25**	**0.974±0.03**
		-	Transcriptomics	0.768±0.08	0.611±0.22	0.33±0.25	0.889±0.03
		DenseNet (169)	Deep Features	0.619±0.06	0.582±0.06	0.347±0.20	0.709±0.08
Histology Subtypes	Sigmoid SVM	ResNet (101)	Deep Radiotranscriptomics	**0.888±0.07**	**0.925±0.04**	**0.743±0.16**	0.933±0.06
		-	Transcriptomics	0.840±0.07	0.906±0.06	0.733±0.22	0.875±0.07
		ResNet (101)	Deep Features	0.763±0.06	0.410±0.13	0.04±0.08	**0.946±0.05**

Table S4. Performance of the SMOTE ML-based pipeline on deep descriptors, transcriptomics and deep radiotranscriptomics analysis. The highest overall score is presented in bold.

Experiments	Classifier	Deep Model (Layers)	Feature type	ACC	AUC	SN	SPC
EGFR	Decision Tree	ResNet (50)	Deep Radiotranscriptomics	**0.805±0.05**	**0.747±0.14**	**0.627±0.33**	**0.869±0.06**
		-	Transcriptomics	0.749±0.06	0.647±0.13	0.453±0.28	0.841±0.07
		ResNet (50)	Deep Features	0.722±0.09	0.557±0.10	0.29±0.20	0.824±0.10
KRAS	RBF-SVM	DenseNet (169)	Deep Radiotranscriptomics	0.728±0.07	**0.736±0.09**	**0.353±0.23**	0.840±0.12
		-	Transcriptomics	**0.743±0.06**	0.542±0.14	0.130±0.17	**0.920±0.08**
		DenseNet (169)	Deep Features	0.717±0.08	0.642±0.11	0.307±0.27	0.849±0.14
Histology Subtypes	k-NN	MobileNet	Deep Radiotranscriptomics	0.838±0.02	**0.908±0.05**	**0.600±0.0**	0.909±0.03
		-	Transcriptomics	**0.839±0.07**	0.729±0.05	0.400±0.20	0.970±0.03
		MobileNet	Deep Features	0.786±0.0	0.567±0.02	0.09±0.07	**1.000±0.0**

Table S5. The most significant transcriptomic features.

Transcriptomics		
EGFR	***KRAS***	**Histology Subtypes**
ASAP3	BBS2	CLSTN1
C1orf52	COQ4	DGKA
CDV3	CUL4A	FRMD8
DAGLB	DLEU2	FYCO1
ENAH	DNAJC7	GOLGA3
FAM160A2	FZD4	HIST1H4H
GOLGA1	LMNA	IGFBP5
GPD1L	MYH9	INPPL1
GSN	PITPNC1	KIF3A
MGLL	PSME2	LEPRE1
RBMX	RASSF4	LIMA1
SIK1	S100A4	PON2
TRAF3	S100A6	PTP4A2
ZDHHC7	SAV1	RNASEH1
ZMYND8	SH3TC1	TPCN1
ZNF318	TROVE2	USP31
	ZNF337	XRCC1

Table S6. The most significant radiomic features.

	Radiomics	
**Experiment**	**Feature**	**Definition**
EGFR	exp_1_original_gldm_DependenceNonUniformityNormalized	measures the similarity of dependence throughout the image, with a lower value indicating more homogeneity among dependencies in the image.
	exp_1_original_glrlm_LongRunHighGrayLevelEmphasis	measures the joint distribution of long run lengths with higher gray-level values
	sq_1_original_glszm_ZoneEntropy	the uncertainty/randomness in the distribution of zone sizes and gray levels. A higher value indicates more heterogeneity in the texture patterns.
	wavelet_bior1.3_1_original_glcm_MCC	The Maximal Correlation Coefficient is a measure of complexity of the texture
	wavelet_bior5.5_1_original_ngtdm_Strength	Strength is a measure of the primitives in an image. Its value is high when the primitives are easily defined and visible, i.e. an image with slow change in intensity but more large coarse differences in gray level intensities
KRAS	sq_1_original_glcm_ClusterShade	Cluster Shade is a measure of the skewness and uniformity of the GLCM. A higher cluster shade implies greater asymmetry about the mean.
	sqrt_1_original_glcm_Idmn	IDMN (inverse difference moment normalized) is a measure of the local homogeneity of an image.
	wavelet_coif5_1_original_glcm_Idmn	
	wavelet_db4_1_original_gldm_LargeDependenceLowGrayLevelEmphasis	Measures the joint distribution of large dependence with lower gray-level values.
	wavelet_rbio2.4_1_original_gldm_LargeDependenceLowGrayLevelEmphasis	
	wavelet_rbio3.1_1_original_gldm_LargeDependenceLowGrayLevelEmphasis	
	wavelet_rbio3.1_1_original_ngtdm_Complexity	a measure of texture complexity, quantifying non-uniformity and sudden changes in intensity values within the region of interest

Histology Subtypes	exp_1_original_glszm_ZonePercentage	measures the coarseness of the texture by taking the ratio of number of zones and number of voxels in the ROI.
	grad_1_original_firstorder_TotalEnergy	Total Energy is the value of Energy feature scaled by the volume of the voxel in cubic mm
	sq_1_original_firstorder_90Percentile	
	sq_1_original_glszm_SizeZoneNonUniformityNormalized	the variability of size zone volumes throughout the image, with a lower value indicating more homogeneity among zone size volumes in the image
	sqrt_1_original_glcm_ClusterShade	
	wavelet_bior3.3_1_original_glcm_ClusterShade	
	wavelet_bior5.5_1_original_glcm_MCC	
	wavelet_bior5.5_1_original_glszm_LargeAreaHighGrayLevelEmphasis	measures the proportion in the image of the joint distribution of larger size zones with higher gray-level values
	wavelet_coif3_1_original_glszm_SmallAreaHighGrayLevelEmphasis	measures the proportion in the image of the joint distribution of smaller size zones with higher gray-level values.
	wavelet_db6_1_original_firstorder_Minimum	
	wavelet_sym4_1_original_gldm_SmallDependenceHighGrayLevelEmphasis	measures the joint distribution of small dependence with higher gray-level values.
exp, exponential; sq, square; sqrt, square root; grad, gradient; bior, biorthogonal; rbio, reverse biorthogonal; coif, coilet; db, daubechies; sym, symlet; GLCM, gray-level covariance matrix; GLDM, gray-level dependence matrix; GLRLM, gray-level run length matrix; NGTDM, neighborhood gray-tone difference matrix; MCC, maximal correlation coefficient; IDMN, inverse difference moment normalized;		

Figures

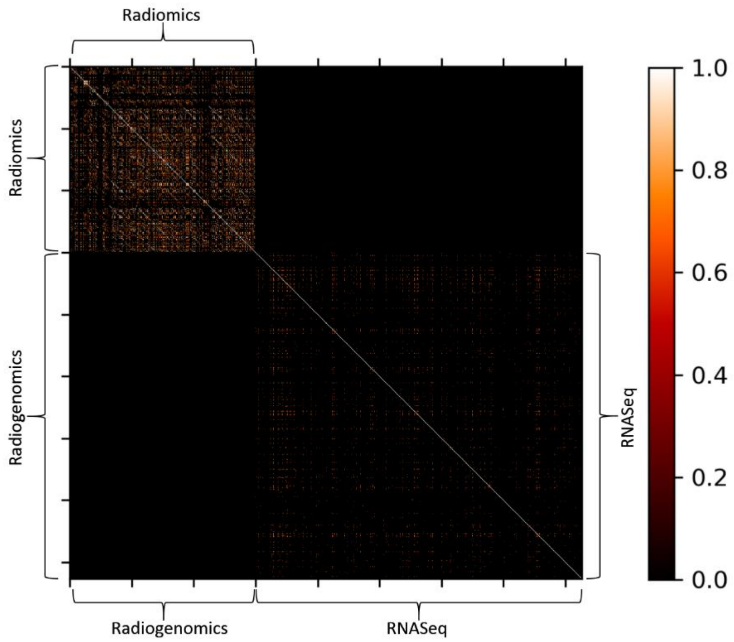

Figure S1. Spearman's correlations (rho>0.6) among radiomic and transcriptomic features. No statistically significant radiogenomic correlations were found.

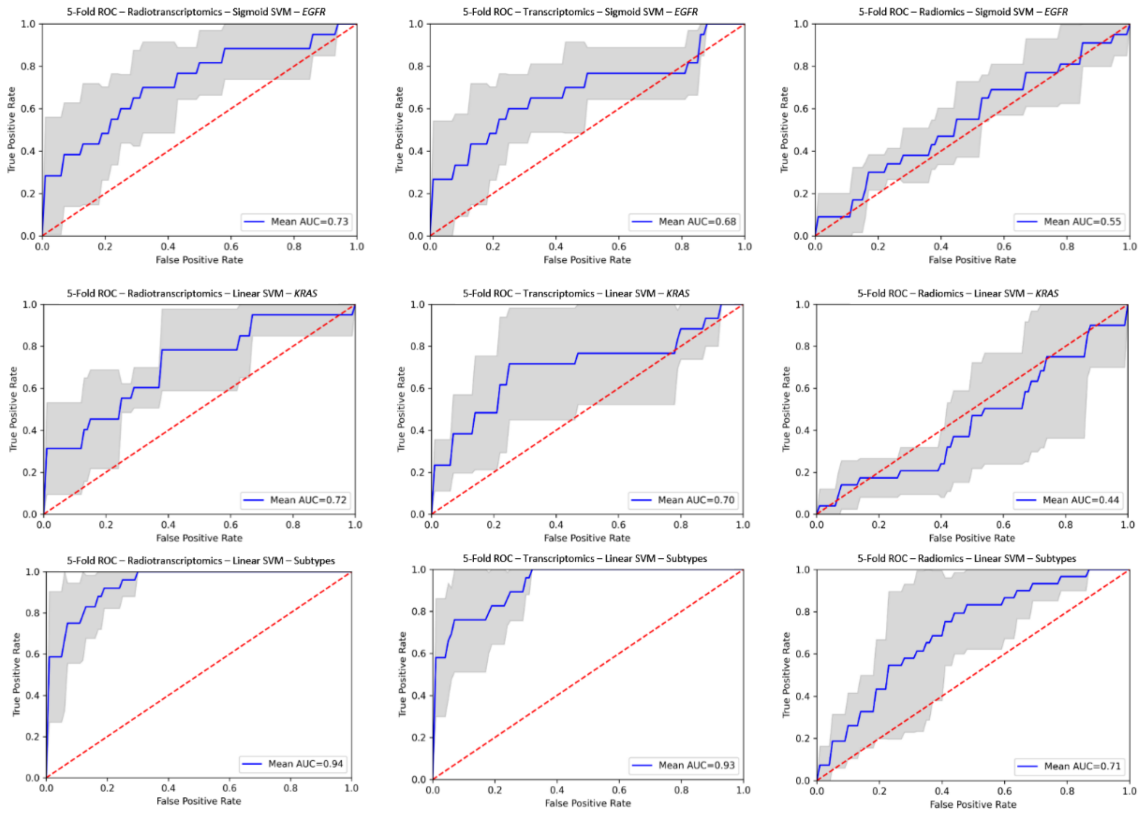

Figure S2. ROC curves for radiotranscriptomics (left column), transcriptomics (center column) and radiomics-based (right column) analysis. The top row represents EGFR (SMOTE), the middle row is KRAS and the bottom row is histology subtypes. The gray region represents the prediction variability among the unseen testing folds. AUC, area under curve; ROC, receiver operating characteristic; EGFR, epidermal growth factor receptor; KRAS, kristen rat sarcoma; SVM, support vector machine; SMOTE, synthetic minority oversampling technique.

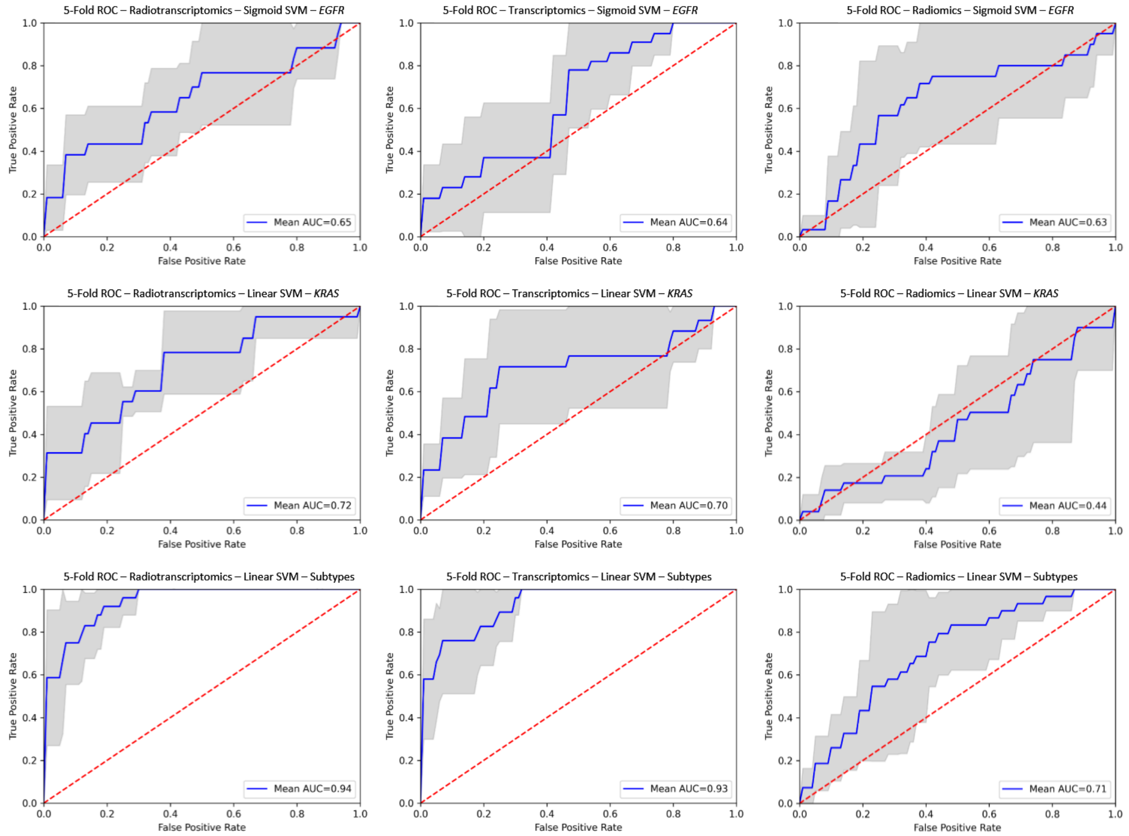

Figure S3. ROC curves for radiotranscriptomics (left column), transcriptomics (center column) and radiomics-based (right column) analysis. The top row represents EGFR, middle row KRAS and the bottom row histology subtypes.

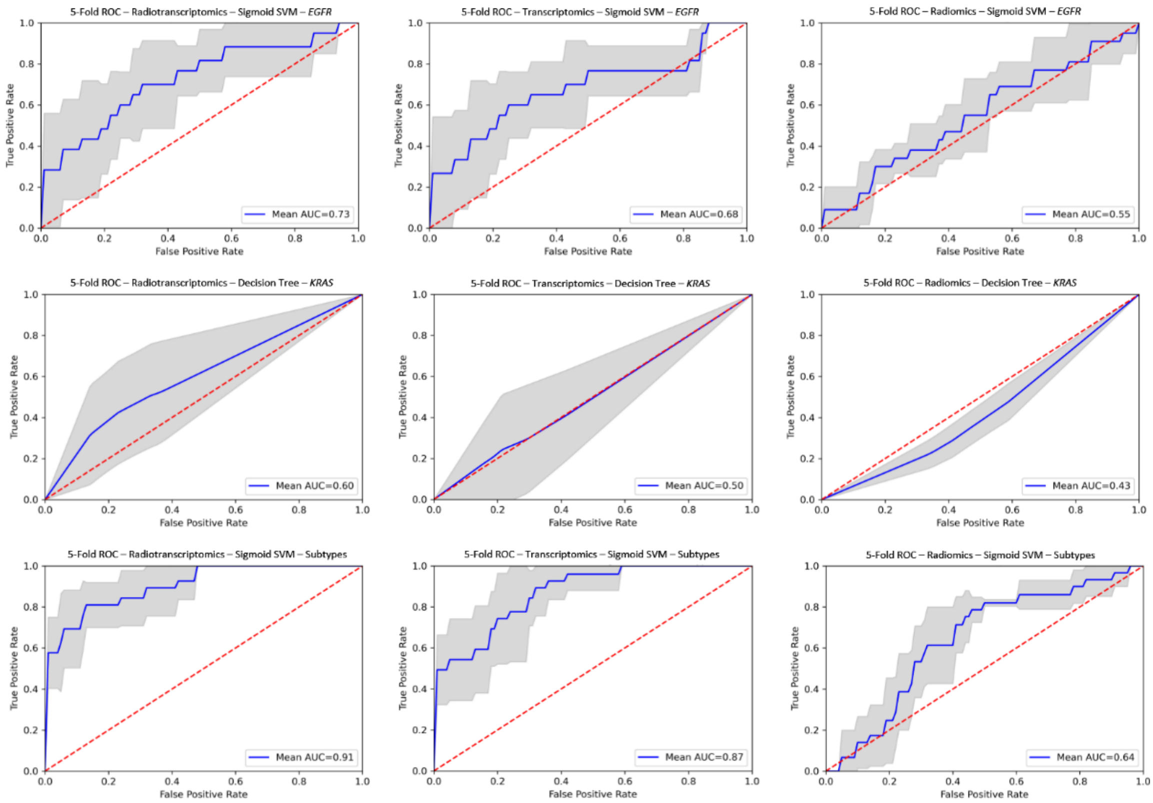

Figure S4. ROC curves for SMOTE radiotranscriptomics (left column), transcriptomics (center column) and radiomics-based (right column) analysis. The top row represents EGFR, middle row KRAS and the bottom row histology subtypes.

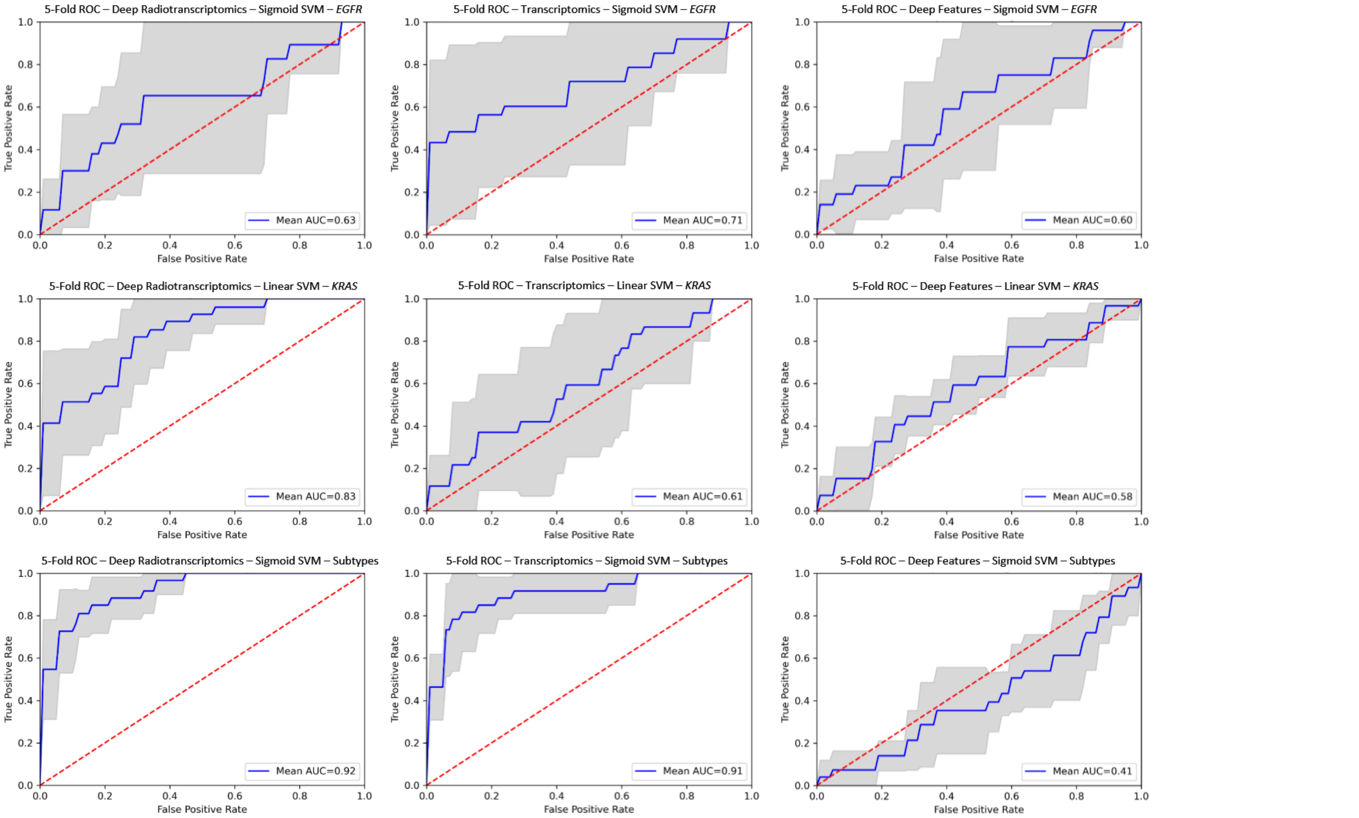

Figure S5. ROC curves for deep radiotranscriptomics (left column), transcriptomics (center column) and deep descriptor-based (right column) analysis. The top row represents EGFR, middle row KRAS and the bottom row histology subtypes.

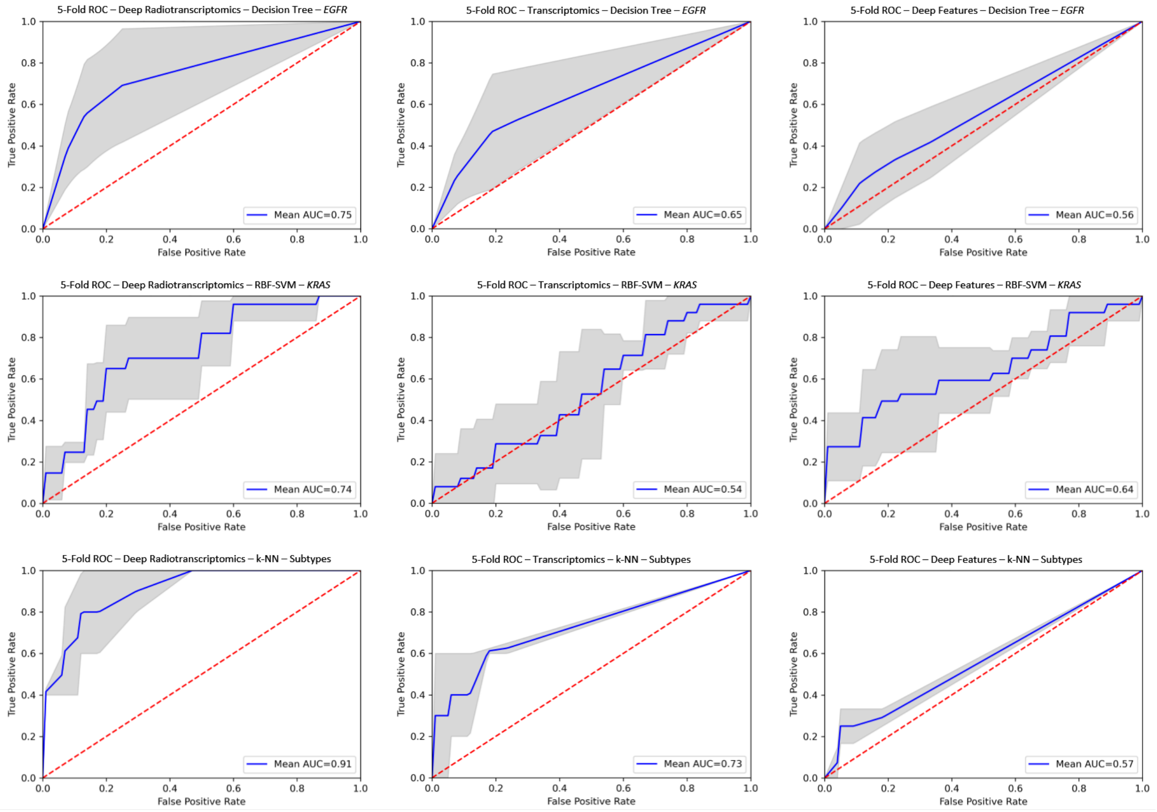

Figure S6. ROC curves for SMOTE deep radiotranscriptomics (left column), transcriptomics (center column) and deep descriptor-based (right column) analysis. The top row represents EGFR, middle row KRAS and the bottom row histology subtypes.
